# Commentary on: The Impact of Hypothermic Oxygenated Perfusion (HOPE) on Immune Responses and Sterile Inflammation in a Preclinical Model of Pancreatic Transplantation by Mesnard B et al. *Transplantation Direct* 2025; 11:e1743; doi: 10.1097/TXD.0000000000001743

**DOI:** 10.1097/TXD.0000000000001842

**Published:** 2025-07-24

**Authors:** Arnau Panisello-Rosello, Carlos Palmeira, Teresa Carbonell, Joan Rosello-Catafau

**Affiliations:** 1 Translational Research and Innovation in Surgery Group (GITIC), Department of General and Digestive Tract Surgery, La Paz University Hospital–IdiPAZ, Paseo de la Castellana, Madrid, Spain.; 2 Centre for Neuroscience and Cell Biology (CNC-CIBB), University of Coimbra, Rua Larga, Coimbra, Portugal.; 3 Department of Cell Biology, Physiology and Immunology, Faculty of Biology, University of Barcelona, Barcelona, Spain.; 4 Department of Experimental Pathology, Steatohepatitis and Liver Transplantation Group, August Pi i Sunyer Biomedical Research Institute (IDIBAPS), Barcelona, Spain.

The intriguing study by Mesnard et al, published in *Transplantation Direct*,^1^ explores the impact of hypothermic oxygenated perfusion (HOPE) on immune responses and sterile inflammation in a preclinical model of donation after circulatory death pancreatic transplantation. The authors clearly describe the suitability of HOPE in reducing endothelial activation compared with static cold storage (SCS), highlighting the relevance of this technique for improving pancreas preservation.

However, from our perspective, it is noteworthy that in this study, the perfusion solution used is institut georges lopez-1 (IGL 1) instead of the traditional Belzer UW machine perfusion solution. The main distinction between these 2 solutions lies in their oncotic agents—polyethylene glycol (PEG) 35 in IGL 1^2^ and hydroxyethyl starch (HES) in Belzer UW machine perfusion solution^2^—as well as in the lower viscosity of the former.^2^ These properties of PEG 35 solutions (IGL 1 and IGL 2), observed in SCS, in our view play a crucial role in improving HOPE conditions, particularly in protecting the superficial endothelial glycocalyx directly exposed to fluid dynamics.^3,4^

The results reported by Mesnard et al^1^ align with the well-established benefits of HOPE strategies for enhancing mitochondrial and glycocalyx protection during SCS. They are even more decisive in preventing the deleterious effects of fluid dynamics and shear stress that lead to endothelial glycocalyx deterioration during HOPE dynamic perfusion.^4,5^ In this regard, the low viscosity of IGL 1 is a contributing factor that helps reduce the inherent shear when this solution is used.

Furthermore, electron microscopy findings reinforce the protective role of PEG 35 in preserving vascular endothelial during static preservation. Electron microscopy reveals that fluorescent PEG 35 effectively deposits onto the vascular bed surface,^3^ forming a protective layer that mitigates endothelial glycocalyx deterioration, promotes NO production, and maintains cellular signal transduction during HOPE when PEG 35 solutions are used.^3,4^ In this sense, syndecan 1 and heparan sulfate levels could be useful markers for evaluating glycocalyx protection and its relationship with NO activity when pancreas specimens are subjected to HOPE for transplantation purposes.

Collectively, this suggests that PEG 35–based solutions such as IGL 1 (and IGL 2) could be suitable for enhancing pancreas NO-mediated protection, potentially improving transplantation outcomes. However, further research is ongoing to evaluate advancements in PEG 35–based solution formulations aimed at optimizing the balance between viscosity reduction and HOPE-mediated endothelial glycocalyx protection in pancreatic transplantation.
